# Child psychiatry services in Asia: evolving state of affairs?

**DOI:** 10.1186/s13034-015-0044-9

**Published:** 2015-05-13

**Authors:** Choon Guan Lim, Benedetto Vitiello

**Affiliations:** Department of Child and Adolescent Psychiatry, Institute of Mental Health, 10, Buangkok View, Hougang, Singapore 539747 Singapore; Treatment and Preventive Intervention Research Branch, National Institute of Mental Health, Bethesda, Maryland USA

Today, there is general agreement among medical professionals that the use of evidence-based medicine is the standard of care when providing medical services, including in child and adolescent psychiatry. Clinical practice should be based on the best available scientific research. Thus, sharing of scientific reports is a critical aspect for proving quality mental health care to children and adolescents. The most influential scientific publications with wide international accessibility are published in English, and much of these publications report on research conducted in Western populations, with more than half coming from the United States and United Kingdom [1]. Yet Asia is the largest continent and houses 70% of the world’s population. China and India, the two most populous countries in the world, alone house 55% of the world’s population. These literature is highly relevant to mental health care in Asia, as it is now generally recognized that there more similarities than differences in clinical presentation and needs across societies and cultures [2]. For example, recent data indicate that the use of psychotropic medications has substantially increased also among Chinese children, and not only in American populations [3]. However, this does not mean that there is little research in child and adolescent psychiatry Asia, but much of this is published in different languages which limit their reach internationally. Encouragingly, journals such as the Asian Journal of Psychiatry and ASEAN Journal of Psychiatry have helped to promote more English publications from the region.

However, there are evident cultural, economic, and organizational differences both within Asia and between Asia and Western countries. These characteristics influence the way mental health is defined, diagnosed, and treated. For example, together with mainstream Western-like medicine, there still exist other medicinal systems within Asia, which are sometimes loosely termed as traditional medicine. One example is traditional Chinese medicine which has been practiced for over two thousand years. However, some treatments approaches may not have been researched in scientific trials considered being of 'good quality' by international standards, and many of these studies would have been published in non-English journals. A recent systematic review attempted to explain for example, how attention deficit hyperactivity disorder (ADHD), one of the most prevalent child psychiatric conditions, was conceptualized in Chinese medicine and highlighted the relatively widespread use of traditional Chinese medicine in Asia for its treatment, for reasons such as cultural acceptability and perceived relative safety compared to Western medications [4]. There should therefore be more effort to focus on conducting research on Asian populations and also on potentially useful traditional medication preparations.

Asia has seen socio-political instability resulting from armed conflicts especially with World War II and the Vietnam War. By the 1980’s, many countries have seen relatively recent stability with resultant significant economic growth. This has led to rapid urbanization and improved living standards, along with access to education and increasing adoption of Western lifestyle habits. However, Asia has varied cultures among its nations, many of which have deep-rooted multi-generation traditions. This interface of differing cultures can influence the presentation and acceptability of mental health services. Even in urbanized Singapore, which has gone from developing to a developed economy in a span of a few decades, there is still significant preference to seek traditional or spiritual healers for mental illness [5]. Access to child and adolescent psychiatry services also vary across Asian countries, and within huge nations themselves such as China and India. Rates of child psychiatric disorders do change over time as well [6]. The organization, accessibility and inter-ministerial collaboration of government medical, social and educational sectors also greatly influence how child psychiatry services are delivered.

There is therefore a need to understand better how child and adolescent mental health care is organized and provided in Asia. This thematic series provides an important opportunity for invited Asian child psychiatrists to summarize important research that has been done in their region, as well as discuss the state of child and adolescent psychiatry services in their respective countries. Drs. Malhotra and Padhy present the general approach to child and adolescent mental illness in India, and discuss the challenges of providing care in the current context. Dr. Zheng provides an overview of pediatric mental health in China and offers perspectives for future developments. Dr. Lim and Colleagues illustrate the developmental of the current mental health services system in Singapore and critically examine both accomplishments and challenges. It will be interesting to compare the impact of the different socio-economic development and cultures on the development of child and adolescent psychiatry services in China, India and Singapore, and learn how each manages the complex task of improving care. It is the intention of this Journal to provide an ongoing forum for presenting and discussing different approaches to child and adolescent mental health across the world.
